# Medical Treatment of Postendoscopic Submucosal Dissection Phlegmonous Gastritis in an Elderly Diabetic Woman with Myelodysplastic Syndrome

**DOI:** 10.1155/2018/8046817

**Published:** 2018-09-30

**Authors:** Ko Matsuura, Shinsuke Hiramatsu, Rika Taketani, Kohei Ishibashi, Masanao Uraoka, Shinichi Baba, Akihiro Nakamura, Hiroshi Takihara, Chie Ueda, Taro Inoue

**Affiliations:** Department of Gastroenterology, Kishiwada Tokushukai Hospital, 4-27-1 Kamori-cho, Kishiwada, Osaka 596-0042, Japan

## Abstract

Phlegmonous gastritis is a rare, suppurative disease characterized by full-thickness exudative changes, infiltration of inflammatory cells, and edema primarily in the submucosal layer. A 76-year-old woman with type 2 diabetes and myelodysplastic syndrome underwent endoscopic submucosal dissection (ESD) for early gastric cancer. Postoperatively, she developed persistent fever and computed tomography displayed full-circumference thickening of the gastric wall and increased levels of fat stranding. Endoscopy showed post-ESD ulcer floor expansion, formation of a false lumen between the ulcer floor and surrounding folds, and adhesion of purulent matter.* Klebsiella pneumoniae*,* Pseudomonas aeruginosa*, and* Candida albicans* were detected from pus culture and* Klebsiella pneumoniae* from blood culture, leading to a diagnosis of phlegmonous gastritis. Contrast examination showed no leakage outside the gastric wall; therefore, the patient fasted and was given antibiotics. She was successfully treated with medical therapy, as demonstrated by repeat endoscopy. Based on our experience, we recommend antibiotics before and after ESD in patients thought to be at high risk of infection, as well as careful postoperative management including postoperative endoscopy.

## 1. Introduction

Phlegmonous gastritis is a rare, suppurative disease characterized by full-thickness exudative changes, infiltration of inflammatory cells, and edema primarily in the submucosal layer. Herein, we report a case of phlegmonous gastritis that occurred after endoscopic submucosal dissection (ESD) for early gastric cancer in an elderly diabetic woman with myelodysplastic syndrome (MDS), treated successfully with medical therapy.

Gastrointestinal phlegmon is common in the appendix but, in rare cases, can affect the esophagus, stomach, duodenum, small intestine, and colon. Phlegmonous gastritis can lead to critical outcomes including sepsis and shock [1]. Phlegmonous gastritis is categorized into 3 groups based on etiology: primary, secondary, and idiopathic [2].

## 2. Case Presentation

A 76-year-old Japanese woman with type 2 diabetes was referred to our hospital for further investigation of anemia. An upper endoscopy to exclude gastrointestinal bleeding demonstrated an IIc+IIa lesion in the antrum. Tubular adenocarcinoma was diagnosed via biopsy and ESD was planned. Initial blood tests showed pancytopenia with white blood cells (WBC) 1,500/*μ*L, hemoglobin (Hb) 4.6 g/dL, and platelets 5.1×10^4^/*μ*L. Serum biochemistry was normal except for HbA1c 7.0%.

On admission, the patient was diagnosed with MDS by the hematology team. She was given transfusions and follow-up was arranged. After a preoperative transfusion, repeat blood tests showed Hb 8.3 g/dL and platelets 23.4×10^4^/*μ*L. ESD was performed due to this improvement and the patient's desired treatment. The patient was subsequently diagnosed with pseudothrombocytopenia based on repeat platelet count levels.

**Figure 1:**
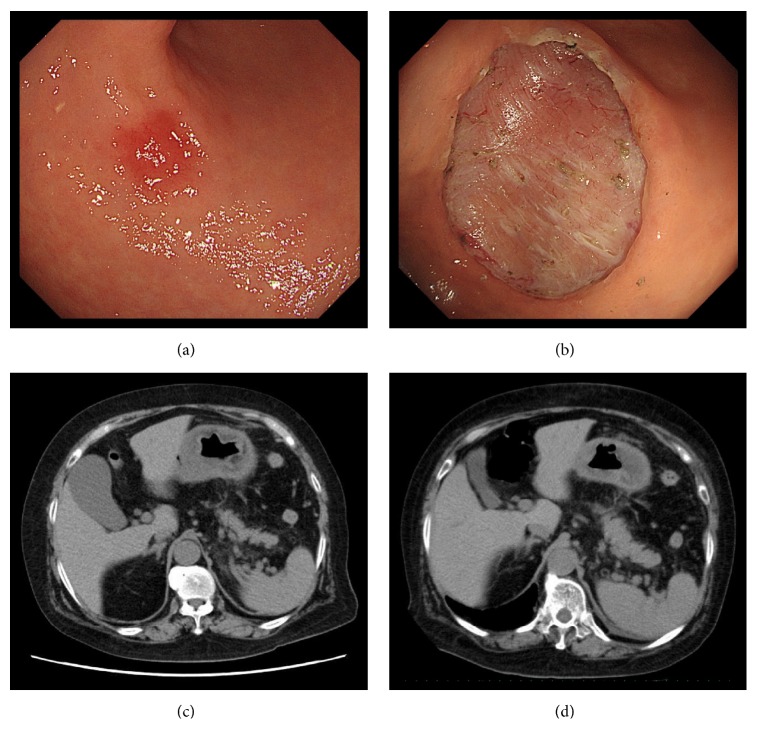
(a, b) ESD. (c) Abdominal CT (day 3 post-ESD). (d) Abdominal CT (day 20 post-ESD).

ESD (Figures 1(a) and 1(b)): There was an IIc+IIa lesion in the antrum. There was only a small amount of intraoperative bleeding and the resection took 80 minutes. The size of the mucosa resected en bloc was 38×34 mm^2^ in diameter including 11×10 mm^2^ of cancer lesion. No perforation and minimal bleeding were observed after finishing ESD.

**Figure 2:**
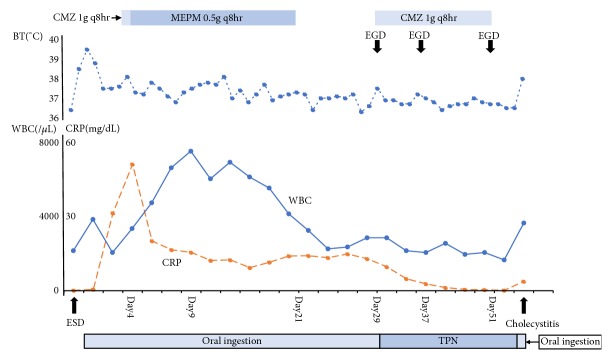
Post-ESD course. Transition of WBC, CRP, body temperature, and course of treatment.

**Figure 3:**
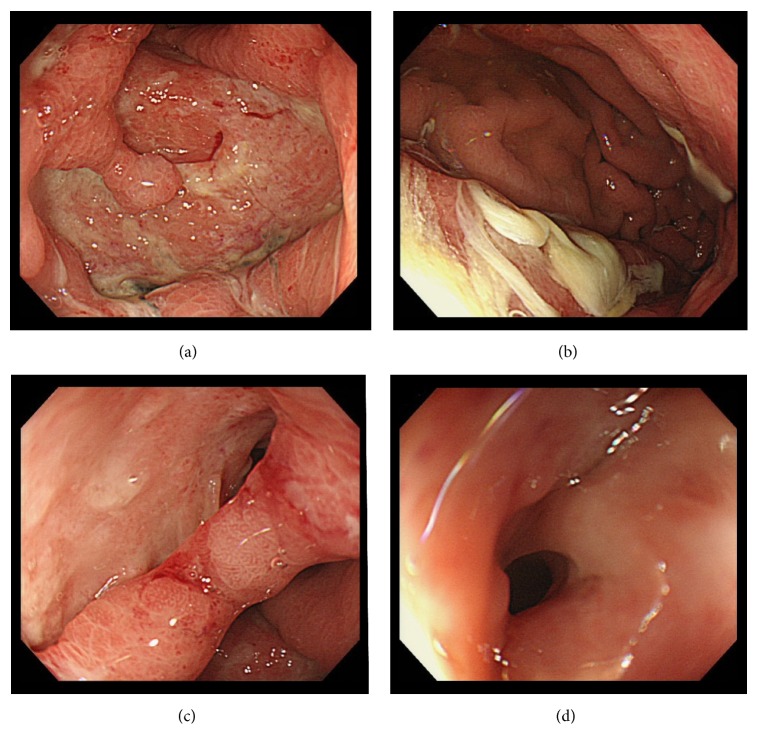
Upper endoscopy (day 29 post-ESD), (a) ulcer, (b) yellow mucus, and (c, d) ulcer and false lumen.

Post-ESD course (Figure 2): No bleeding was observed on repeat endoscopy performed the day after ESD; therefore, the patient was allowed to eat. Her temperature spiked to around 38°C that day, and post-ESD abdominal computed tomography (CT) was performed on day 3 (Figure 1(c)), which showed full-circumference thickening of the gastric wall. However, the patient did not exhibit abdominal pain, so a definitive diagnosis of phlegmonous gastritis could not be concluded. Antibiotic treatment with cefmetazole sodium (CMZ, 3 g/d) was started to cover for* E. coli* urinary tract infection with a positive urine culture. However, after* Klebsiella pneumoniae* was detected in a blood culture, the possibility of a bloodstream infection related to ESD injection was considered. C-reactive protein (CRP) levels increased to 51.5 mg/dL; therefore, antibiotic treatment was switched to meropenem hydrate (MEPM, 1.5 g/d) on day 4 post-ESD, after which the inflammatory reaction gradually improved. Abdominal CT performed on day 20 post-ESD (Figure 1(d)) showed full-circumference thickening of the gastric wall and increased levels of perigastric fat stranding. At this time, the patient had no high fever and abdominal pain; therefore, antibiotic treatment was terminated. On day 29 post-ESD, upper endoscopy was performed (Figure 3) because the patient began experiencing epigastric pain after eating on day 27 post-ESD. In addition to a post-ESD ulcer in the antrum, we observed expansion of the ulcer floor, a false lumen between the ulcer floor and surrounding folds on the lesser curvature side, and yellow mucus thought to be purulent matter adhered to the superior portion of the gastric body. Based on these findings, a diagnosis of phlegmonous gastritis was made. Contrast examination with gastrografin did not show any exudate outside the gastric wall from the false lumen.

**Figure 4:**
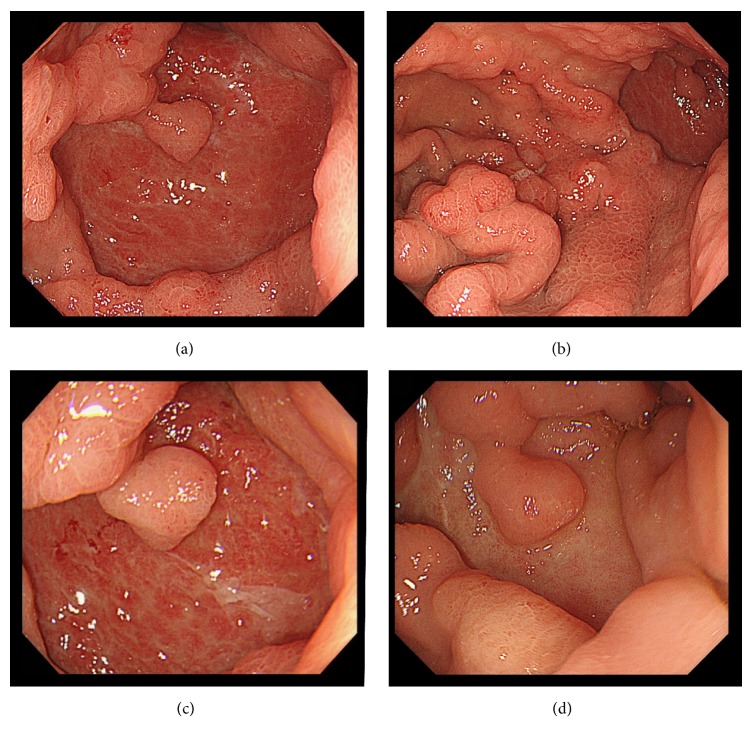
(a) day 37 post-ESD, (b) day 37 post-ESD, (c) day 51 post-ESD, and (d) 4 months post-ESD.

Therefore, antibiotic treatment was continued.* Klebsiella pneumoniae*,* Pseudomonas aeruginosa*, and* Candida albicans* were detected from a pus culture. The patient was made nil by mouth, given intravenous hyperalimentation, and was treated with antibiotics (CMZ 3 g/d) and proton-pump inhibitors (PPI). Endoscopy performed on day 37 post-ESD (Figures 4(a) and 4(b)) showed granulation on the ulcer floor. Endoscopy performed on day 51 post-ESD (Figure 4(c)) showed that the ulcer shrunk and the false lumen disappeared and, on the basis of these findings, antibiotic treatment was terminated. The phlegmonous gastritis was considered to have been cured with medical therapy. The patient resumed eating on day 55 post-ESD, and acute cholecystitis occurred the next day. She underwent cholecystectomy which was complicated by a postoperative wound infection and intra-abdominal abscess, which improved with antibiotic therapy. She was discharged from the hospital on week 16. Endoscopy at 4 months post-ESD (Figure 4(d)) showed complete resolution.

## 3. Discussion

Herein, we describe a patient with a history of MDS and diabetes who developed phlegmonous gastritis after ESD for early gastric cancer.

Abdominal CT, upper endoscopy, and EUS are considered useful diagnostic tools. CT can be used to observe full-circumference thickening of the gastric wall, which if accompanied by severe inflammatory findings could indicate phlegmonous gastritis, though it is often difficult to reach a definitive diagnosis by CT alone [3]. In the present case, the above findings were observed by CT on days 3 and 20 post-ESD, but because of the absence of abdominal pain, we were unable to confirm a diagnosis of phlegmonous gastritis at this time. Endoscopic findings include rubor, edema, and erosion [4].

Searching PubMed for the keywords “endoscopic submucosal dissection” and “phlegmonous gastritis” in papers published between 1983 and 2017, we identified only 1 case report on post-ESD phlegmonous gastritis [5]. Total gastrectomy was performed in the case, but we treated our patient with medical therapy.

The most common pathogenic bacterium in phlegmonous gastritis is* Streptococcus* at 60-70%, with* Enterococcus, Klebsiella pneumoniae, Staphylococcus, Haemophilus influenzae, and Clostridium* also being reported [1].

Phlegmonous gastritis is caused by reduced immunity because of factors such as habitual alcohol use and diabetes in about half of patients [1]. Our patient also had MDS, which was implicated in the etiology of phlegmonous gastritis. The present case is the only one we could find that occurred post-ESD and which was accompanied by positive blood cultures.

Elevated gastric fluid pH is also said to be a risk factor for phlegmonous gastritis [6]. The patient in the present case had been taking a PPI (lansoprazole 15 mg/d) and was also administered PPI intravenously before and after ESD. It is therefore possible that administration of a strong acid-suppressive agent reduced the stomach's ability to kill bacteria by increasing gastric pH, which contributed to onset of the disease [6]. PPIs are said to be useful in healing post-ESD ulcers [7], but given their association with phlegmonous gastritis, further studies are warranted to investigate whether PPIs or H2-blockers are indicated in immunocompromised patients.

In a 1919 review of 215 cases of phlegmonous gastritis, Sundberg et al. reported a mortality rate of 92% [8], while in a 2014 review of 45 cases of phlegmonous gastritis, Rada-Palomino et al. reported a mortality rate of 27%, showing that prognostication improved over time [1]. It has also been reported that antibiotic therapy can be selected as long as there are no serious complications, such as hemorrhage or perforation [4], and that surgery and antibiotic therapy do not produce significantly different outcomes [1]. The present case was not accompanied by perforation or hemorrhage, and because of the presence of MDS and diabetes, we decided that the patient was susceptible to infection and postoperative suture rupture, so we elected for antibiotic treatment.

Gastric ESD was first reported in 1998 and became eligible for insurance coverage in 2006. This short history and lack of case reports on post-ESD phlegmonous gastritis mean that much remains unclear about its incidence rate, relationship with the techniques used, and other factors [5]. It is thought that this disease can be caused by a variety of pathogenic bacteria, so starting therapy with a broad-spectrum antibiotic then changing to a narrower range based on reliable culture results appears to be a valid approach. Different bacteria are often detected from pus, gastric mucosa biopsies, blood, and other sources, so it must be recognized that culture results are not absolute and there should be no hesitation to use broad-spectrum antibiotics for long periods, depending on the clinical course. Antibiotics are not routinely administered before and after gastric ESD [9], but in patients like ours with high infection risk factors, such as high age, diabetes, and habitual alcohol use, the possibility of postoperative phlegmonous gastritis should be taken into consideration when deciding whether to administer antibiotics before and after surgery. Such patients also require careful postoperative management, including upper gastrointestinal endoscopy.
